# Molecular Recognition of Arginine by Supramolecular Complexation with Calixarene Crown Ether Based on Surface Plasmon Resonance

**DOI:** 10.3390/ijms12042315

**Published:** 2011-04-04

**Authors:** Hongxia Chen, Limin Gu, Yongmei Yin, Kwangnak Koh, Jaebeom Lee

**Affiliations:** 1 School of Life Sciences, Shanghai University, Shanghai 200444, China; E-Mail: hxchen@shu.edu.cn (H.C.); 2 Henan Chemical Industry Research Institute Co. Ltd., Zhengzhou 450052, China; E-Mail: glm221@sohu.com; 3 Department of Oncology, Jiangsu Province Hospital, Nanjing 210029, China; 4 College of Nanoscience and Nanotechnology, Pusan National University, Pusan 609-735, Korea; E-Mails: koh@pusan.ac.kr (K.K.); jaebeom@pusan.ac.kr (J.L.)

**Keywords:** arginine, calixarene crown ether, amino acid, self-assembled monolayer (SAM), surface plasmon resonance (SPR)

## Abstract

Arginine plays an important role in cell division and the functioning of the immune system. We describe a novel method by which arginine can be identified using an artificial monolayer based on surface plasmon resonance (SPR). The affinity of arginine binding its recognition molecular was compared to that of lysine. In fabrication of an arginine sensing interface, a calix[4]crown ether monolayer was anchored onto a gold surface and then characterized by Fourier Transform infrared reflection absorption spectroscopy, atomic force microscopy, and cyclic voltammetry. The interaction between arginine and its host compound was investigated by SPR. The calix[4]crown ether was found to assemble as a monolayer on the gold surface. Recognition of calix[4]crown monolayer was assessed by the selective binding of arginine. Modification of the SPR chip with the calix[4]crown monolayer provides a reliable and simple experimental platform for investigation of arginine under aqueous conditions.

## 1. Introduction

Arginine, one of the 20 natural amino acids, plays an important role in cell division, healing of wounds, removal of ammonia from the body, functioning of the immune system, and release of hormones [1–3]. Arginine, combined with proanthocyanidins [4] or yohimbine [5], has also been used for treating erectile dysfunction. Arginine is the immediate precursor of NO, urea, ornithine, and agmatine [6–8]. It is considered a sign of a healthy endothelium. As a charged amino acid, arginine is the best target among the twenty amino acids for the molecular recognition of a specific side chain in a peptide [9,10]. The different chemical functionalities of the basic side chains enhance the possibility of achieving specificity. Thus, an assessment of the molecular recognition of arginine is of great importance in biochemical studies. In related gas-phase work, Freiss and Zenobi have utilized a series of sulfonates for the molecular recognition of arginine [11]. Molecular recognition is possible primarily because of the electrostatic attraction between the basic guanidinium group of arginine and acidic disulfonate. In addition, several molecules were designed for the assessment of the molecular recognition of arginine in solutions. Dougherty and co-workers have synthesized a cyclophane-based host that forms a stable complex with arginine with the aid of a combination of hydrophobic, cation-π, and ion-pairing interactions [12]. Schrader and co-workers have developed a series of bisphosphonate receptors that utilize a combination of hydrogen bonding, electrostatic interactions, and cation-π effects to recognize alkyl guanidinium groups [13,14]. Bell and co-workers have developed the “arginine cork”, which demonstrates arginine selectivity through the formation of several specific hydrogen bonds accompanied by electrostatic attraction [15].

It is well-known that calix[n]arenes are used for the molecular recognition of ions, amino acids, hormones, sugars, peptides, nucleic acids, and proteins, which are fundamental substrates in biological and artificial processes [16–20]. A majority of the studies on calixarenes have focused on the calix[4]arenes, primarily because they possess open and rigid structures that are desirable for molecular recognition [17,19,21]. Arena *et al.* examined the complexation of a number of amino acids with soluble calix[4]arenes derivatized with carboxylic acids and sulfate esters [22]. The host compounds were calix[4]arenes derivatized with l-aminophosphonates which were found to exhibit high selectivity as carriers that transport the zwitterionic forms of aromatic amino acids across membranes [23]. Silica-bonded calixarenes have been used for retaining amino acid esters in chromatography [24]. Recently, the molecular recognition capabilities of 18-crown-6 (18C6) and dibenzo-30-crown-10 ether have been studied for examining their use as a specific host for the side chain of lysine and arginine in the gas phase [9]. Calixarenes and crown ethers are similar in their ability to function as host compounds, forming inclusion complexes with organic amines and metal ions [16]. The calixarenes possess sizable inner “cavities” that crown ethers or their derivatives do not have. Investigations on the use of a combination of calixarenes and crown ethers for amino acid recognition are less common.

To the best of our knowledge, there is no study on arginine recognition with calixarene crown ether. We present a study on the molecular recognition capability of a combination of crown ether and calixarene function groups. A calix[4]crown ether derivative has previously been used as a protein linker [25,26]; the derivative forms a self-assembled monolayer (SAM) on the gold surface, and allows the captured protein to be tightly bound to the linker molecules. The major binding force could be attributed to the ionized amine groups of capture proteins, which bind to the crown moiety of the linker molecule via host-guest interactions. In addition, π-cation interactions between ionized groups of protein and calixarene may be involved in the protein immobilization. Oh *et al.* added extra positively charged arginines to a protein, and demonstrated that the interaction between the capture protein and the calixcrown derivative was increased [26].

The purpose of our research is to develop a novel arginine-recognition interface by using an advanced and simple optical system in order to obtain highly sensitive and simple sensors. To achieve this goal, we employed an SPR system with a recognition interface in order to selectively identify arginine. A self-assembly technique was utilized to construct a well-characterized sensing layer, and then, the monolayer formation process was investigated using SPR spectroscopy. The characterization of the calixcrown ether SAM was monitored by using FT-infrared reflection absorption spectroscopy (FTIR-RAS), an atomic force microscopy (AFM) image, and electrochemical analysis. Interaction between arginine and calixcrown ether monolayer was observed. The change in the surface refractive index for different concentrations of arginine on the monolayer was calculated by simulating the SPR experimental data. Further, to evaluate the usability of calixcrown ether monolayer as an arginine recognition chip, its selective binding with arginine was compared to that with lysine on the basis of SPR results.

## 2. Experimental Section

### 2.1. Chemicals and Reagents

**Figure 1.**
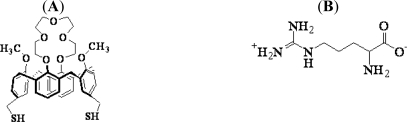

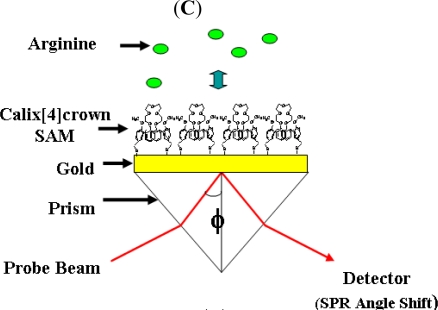
Molecular structure of (**A**) calix[4]crown; (**B**) arginine; (**C**) Schematic diagram of the sensor chip configuration.

Calix[4]crown, one of calix[4]arene derivatives with a crown ether moiety, was purchased from Proteogen (Seoul, Korea) and used as a linker system for the recognition of arginine (Figure 1A). l-arginine (Figure 1B) and l-lysine were purchased from Sigma Chemicals (St. Louis, MO, USA). Amino acid solutions with concentrations in the range of 1 × 10^−7^ to 1 × 10^−3^ M were prepared in phosphate buffer saline (PBS) buffer solution. PBS solution (10×), and other reagents were obtained from Sigma Chemicals (St. Louis, MO, USA). Milli-Q grade (18.2 mΩ cm^−1^) water was used for the preparation of the sample and buffer solutions.

### 2.2. Formation and Characterization of Calixcrown Ether SAM

A microscope cover glass (18 mm × 18 mm × 0.15 mm, with a refractive index of 1.515, Matsunami, Japan) with a gold layer was used as the substrate for the formation of the calix[4]crown SAM. The gold film (thickness ≈ 50 nm) was deposited on the cover glass using a sputter coating system (E5000, Polaron Co., UK) under conditions of 2.0 × 10^−2^ mbar and 20 mA for 135 s. The sputtered gold substrate was first rinsed with distilled water, then with methanol, and finally with acetone. The gold chip was softly dried in a nitrogen stream, and the chip was made ready for use. The calix[4]crown solution was prepared by the mixing chloroform and methanol in the volume ratio of 1:100 (v:v). The SAM was developed by immersing the gold chip in 0.1 mM calix[4]crown solution. The immobilization process was monitored by using SPR spectroscopy. After immobilization, the sensor chip was rinsed with chloroform-methanol mixture solution (1:100, v:v) for 15 min, and then dried under N_2_ stream. The calix[4]crown SAM was carefully characterized by cyclic voltammetry (CV), FTIR-RAS, and AFM. CV measurements were performed on a BAS-100B electrochemical analyzer (Bioanalytical Systems Inc., USA). The three-electrode system used consisted of a Ag/AgCl reference electrode with a filling solution of saturated KCl, a platinum coil as the auxiliary electrode, and gold as a working electrode with a scan rate of 50 mVs^−1^. The FTIR-RAS spectra (Magma-IR TM 550, Nicolet, USA) were measured with a resolution of 2 cm^−1^. The glazing angle was maintained at 80°, and a p-polarized IR beam was used as the light source. AFM images (SPM-LS, Park Scientific Instruments, USA) were collected in the non-contact tapping mode. The silicon nitride cantilevers had a nominal spring constant of about 0.067 Nm^−1^. The scanning parameters were adjusted so that clear images were obtained. Clear images are important to observe the effects of the SAM on the deposited gold surface.

### 2.3. Recognition of Arginine on the Calix[4]Crown Monolayer

A home-made SPR system based on the traditional Kretschmann configuration was used; the system was described in our previous paper [13,14]. Briefly, a laser diode (LD, λ_max_ = 675 nm) was used as the light source. The intensity of light reflected through the polarizer and the prism was measured using a photodiode detector (ANDO Electric Co. Ltd., AQ-1976, Kanagawa, Japan). The incident angle at the prism was varied using a motorized rotary stage and a controller (Suruga Seiki, D80, Shizuoka, Japan). The signal from the photodiode was converted through a signal process board (K-MAC Co., Spectra View 2000, Taejeon, Korea); and could be interfaced with a computer. The angle resolution of the SPR system that was determined by the resolution of the motorized rotary stage was 0.004°.

The sensor chip configuration is shown in Figure 1C. The gold chip modified by calix[4]crown was rinsed with PBS solution. Five different arginine solutions with concentrations in the range of 1.0 × 10^−7^ to 1.0 × 10^−3^ M were prepared in PBS. These solutions were injected into the cell, monitored by SPR, and rinsed with PBS solution to remove reversibly bound amino acid. To compare the recognition selectivity, lysine solutions were flowed onto the sensing monolayer using similar procedures.

## 3. Results and Discussion

### 3.1. Characterization of Calix[4]Crown SAM

**Figure 2.**
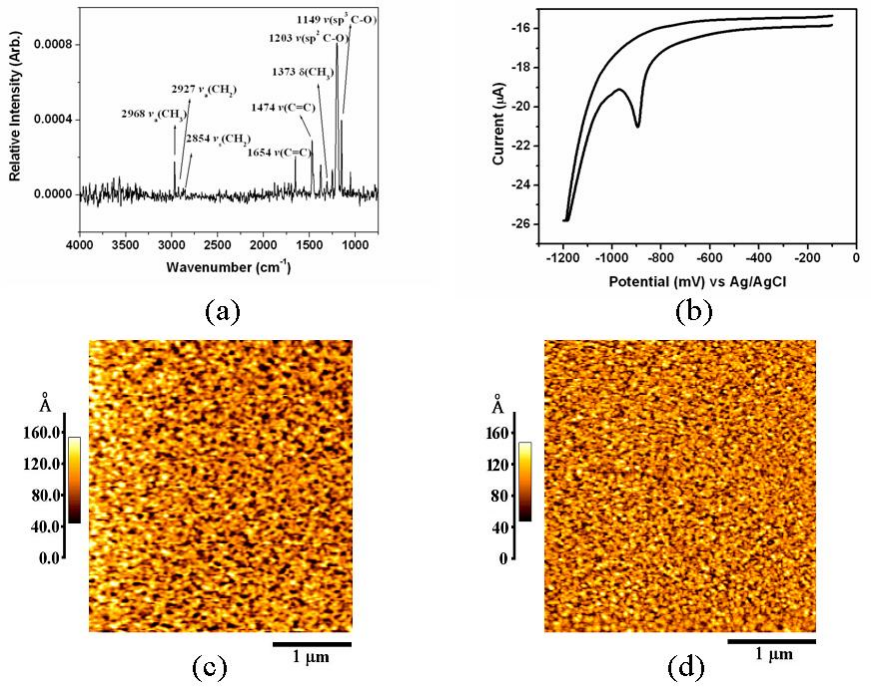
Characterization of the calix[4]crown SAM: (**A**) FTIR-RAS spectrum; (**B**) CV for reduction of the calix[4]crown on the gold electrode in 0.5 M KOH; and AFM image of (**C**) bare gold; and (**D**) the calix[4]crown SAM on gold.

Calix[4]crown possessing a crown-ether moiety acts as a host cavity for amine groups on the protein surface. Previous studies [25,26] have demonstrated that calix[4]crown bound protein molecules tightly, and that its self-assembled monolayer is a very efficient molecular linker system for immobilization of protein on a substrate surface without any loss of protein activity. To construct an arginine chip on a solid substrate, we prepared a calix[4]crown surface on a gold-coated cover glass. A typical FTIR-RAS spectra of calix[4]crown SAM on a gold surface is shown in Figure 2A. In particular, from *ν* (sp^2^ C–O) stretching at 1209 cm^−1^, the presence of the crown ether group on the gold surface was confirmed. The properties of the electrode modified with the calix[4]crown SAM can be estimated by subjecting the electrode to reductive desorption experiments. Figure 2B shows the reductive desorption peak of the calix[4]crown SAM on the gold electrode. This peak has been attributed to the reductive desorption of thiolated compounds that are chemisorbed on gold. Assuming that all thiolated compounds are reduced/oxidized in the CV experiments, the surface coverage can be determined from the CV measurements [27,28]. After accounting for the surface roughness of the gold electrode, the surface coverage of calix[4]crown SAM was calculated to be 2.209 × 10^−10^ mol cm^−2^, which was consistent with the theoretical monolayer value (6.002 × 10^−10^ mol cm^−2^) obtained using CS Chemdraw™ (Cambridgesoft Co., USA). These results suggest that the self-assembled calix[4]crown monolayer can provide favorable conditions that increase the detection resolution of arginine in aqueous solution. The AFM images show the surface geometry of a bare gold surface and the gold surface modified by calix[4]crown (Figure 2C,D). The bare gold surface showed a relatively larger sized domain comparing with the calix[4]crown surface, which was caused by the formation of a self-assembled monolayer of calix[4]crown molecules. From the combination of the results of FTIR-RAS, AFM, and CV, it is clear that calix[4]crown was immobilized as a dense monolayer. This engineered construction of an artificial recognition monolayer on the gold surface was ready to be fabricated as an arginine detection chip.

### 3.2. Recognition of Arginine on the Calix[4]Crown Monolayer

**Figure 3.**
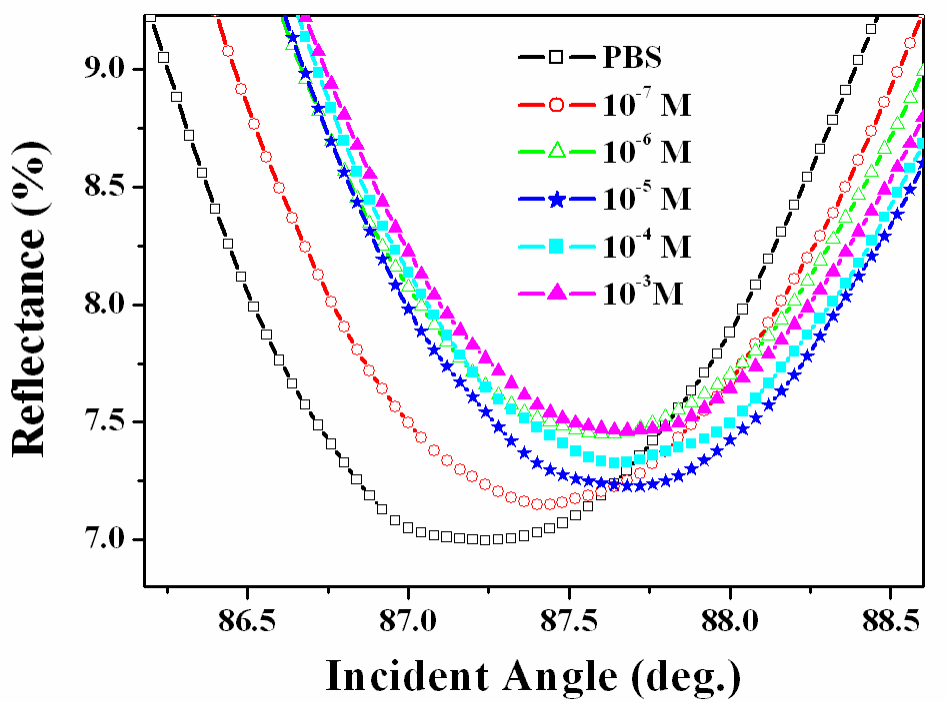
Experimental SPR curves of Au surface modified with calix[4]crown SAM according to the interaction with different concentration of arginine in PBS.

To compare the binding properties, arginine was placed on the surface of the chip modified with the calix[4]crown SAM. As the concentration of arginine increases, the SPR angle shifts that result from the molecular interaction between the calix[4]crown SAM and arginine gradually increases (Figure 3). The interactions between lysine and calix[4]crown were monitored using the same procedure (data not shown).

**Figure 4.**
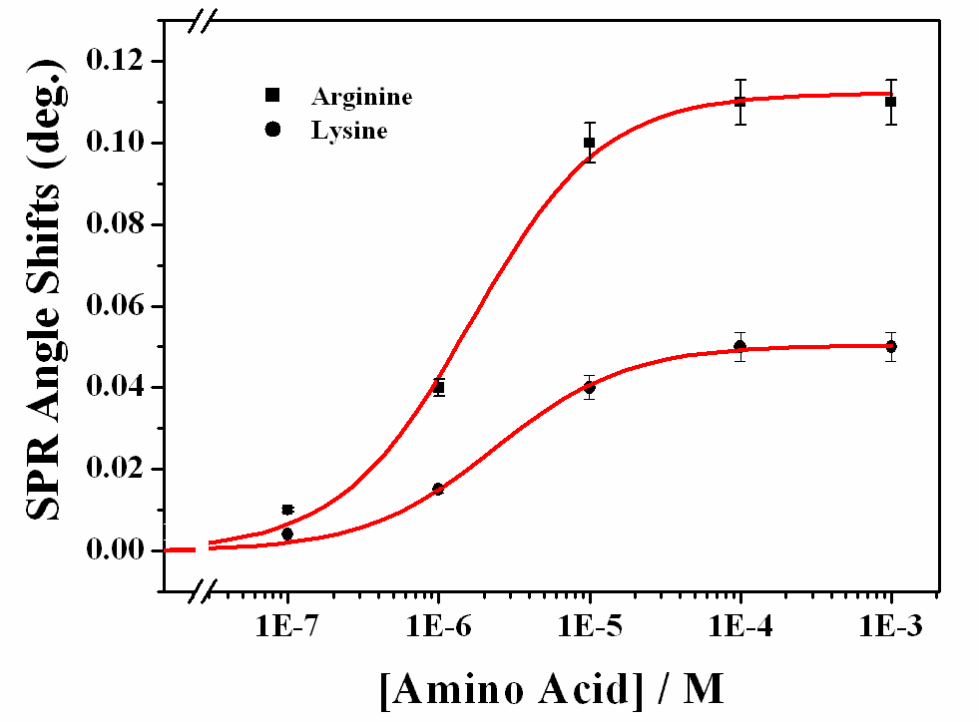
Dose-response curves of sensor chip immobilized by calix[4]crown SAM, represented by SPR angle shifts according to the interaction with arginine (rectangular) and lysine (circle). Solid lines represent the result of one-site binding fitting.

The shift in the SPR angle corresponding to the increase in arginine and lysine concentrations from zero (buffer) to 1 × 10^−3^ M is 0.11°and 0.054°, respectively. The higher SPR response for arginine one-site binding fitting showed that the binding constants for arginine and lysine are 1.63 × 10^−6^ M (R^2^ = 0.997) and 2.38 × 10^−6^ M (R^2^ = 0.998), respectively (Figure 4). The binding of the calix[4]crown SAM to arginine may involve strong hydrogen bonding of the crown-like loop [26]. It is well known that calix[4]arene crown ether binds to and completely encapsulates the cation [29]. Gawley *et al*. presented a possible mode of binding between crown ether and guanidinium, which shows the presence of several hydrogen bonds between the guanidinium and crown ether oxygens [30].

The detection limit for arginine was found to be 1.0 × 10^−7^ M. The selectivity for arginine was greater than that for lysine. Compared to the conventional gas-phase method and chemosensor, this simple arginine detection system based on SPR is quite remarkable [9,11].

SPR is a collective electron excitation that exists at the interface between two media with different dielectric constants. The dipole moment of a molecule is a factor that is measured for determining the charge distribution of the overall molecule; the formation of the calix[4]crown ionic complex with arginine and lysine affected the dipole moments. The higher sensitivity for arginine compared to that for lysine is because of the difference in their structures. The arginine structure has a –CNH_2_NH_2_^+^ (Figure 1B) amine group. The presence of this group increases the polarizability of calixarene-arginine complex, which leads to a strong SPR response.

The dipole moment is closely related to the dielectric constant *κ* through the relation

(1) $$P=(\kappa -1){ɛ}_{0}E$$

where *P* is the total polarization of the sample, *κ* = *ɛ*/*ɛ*_0_ (where *ɛ* and *ɛ*_0_ are the permittivity of the material and the permittivity of vacuum, respectively), and *E* is the local electric field vector. Note that for optical frequencies, the dielectric constant is related to the refractive index *n* as follows:

(2) $$\kappa =n^{2}$$

Therefore, a variation in the dipole moments of arginine- or lysine-derived calix[4]crown SAM results in a variation in the dielectric constant, which consequently results in a distinct change in RI and greater SPR angle shifts (Equations 1 and 2).To further study the RI change on the sensor surface, the RI was determined through four-layer theoretical simulation using the Fresnel equation and SPR experimental data [28]. Table 1 represents the RI changes obtained through computer simulation when arginine was bound on the calix[4]crown SAM. It was observed that RI increased linearly with a gradual change in arginine concentration.

## 4. Conclusions

We have fabricated a simple arginine recognitional platform with a novel artificial calix[4]crown monolayer based on SPR spectroscopy. Calix[4]crown was immobilized on the gold surface and characterized by FTIR-RAS, AFM, and CV. Specific binding of arginine over lysine on this sensing interface is accessible based on the SPR detection system. This approach provides an efficient method for fabrication of a simple arginine detection system. The host–guest binding between calix[4]crown SAM and arginine facilitates the recognition of arginine in the concentration range of 1.0 × 10^−7^ to 1.0 × 10^−4^ M by using SPR. These experimental results will be useful for designing a simple and efficient arginine recognition interface.

## Figures and Tables

**Table 1.** The refractive index (RI) of the interfacial layer corresponding to the interaction between calix[4]crown SAM and various concentrations of arginine.

**[arg.] (M)**	1.0 × 10^−7^	1.0 × 10^−6^	1.0 × 10^−5^	1.0 × 10^−4^	1.0 × 10^−3^
**RI**	1.323	1.365	1.387	1.405	1.406
